# Genetic Diversity of Highly Pathogenic Avian Influenza Viruses Isolated in Hokkaido, Japan, During Winter 2024–2025

**DOI:** 10.3390/pathogens14090951

**Published:** 2025-09-21

**Authors:** Norikazu Isoda, Lim Yik Hew, Kazuki Nishikawa, Fumihito Takaya, Yo Shimazu, Daiki Kobayashi, Kei Nabeshima, Hisako Honjyo, Mana Esaki, Kosuke Okuya, Kosuke Soda, Hiroshi Ito, Asuka Kumagai, Hayate Nishiura, Takahiro Hiono, Hiroki Takakuwa, Tatsufumi Usui, Makoto Ozawa, Yuko Uchida, Manabu Onuma, Yoshihiro Sakoda

**Affiliations:** 1 Laboratory of Microbiology, Department of Disease Control, Faculty of Veterinary Medicine, Hokkaido University, Sapporo 060-0818, Japan; 2 One Health Research Center, Hokkaido University, Sapporo 060-0818, Japan; 3 Hokkaido University Institute for Vaccine Research and Development (HU-IVReD), Hokkaido University, Sapporo 001-0020, Japan; 4 International Collaboration Unit, International Institute for Zoonosis Control, Hokkaido University, Sapporo 001-0020, Japan; 5 Botanic Garden, Field Science Center for Northern Biosphere (FSC), Hokkaido University, Sapporo 060-0003, Japan; 6 Ecological Risk Assessment and Control Section Center for Environmental Biology and Ecosystem, National Institute for Environmental Studies, Tsukuba 305-8506, Japan; 7 Joint Graduate School of Veterinary Science, Kagoshima University, Kagoshima 899-4101, Japan; 8 Department of Pathogenetic and Preventive Veterinary Science, Joint Faculty of Veterinary Medicine, Kagoshima University, Kagoshima 899-4101, Japan; 9 Kagoshima Crane Conservancy, Izumi, Kagoshima 899-0208, Japan; 10 Avian Zoonosis Research Center, Faculty of Agriculture, Tottori University, Tottori 680-0853, Japan; 11 Emerging Virus Group, Division of Zoonosis Research, National Institute of Animal Health, National Agriculture and Food Research Organization, Tsukuba 305-0856, Japan; 12 Faculty of Life Sciences, Kyoto Sangyo University, Kyoto 603-8555, Japan; 13 Transboundary Animal Diseases Research Center, Joint Faculty of Veterinary Medicine, Kagoshima University, Kagoshima 899-4101, Japan

**Keywords:** clade 2.3.4.4b, genetic and antigenic analysis, high pathogenicity avian influenza, Japan, winter of 2024–2025

## Abstract

Genetic and antigenic analyses were performed on highly pathogenic avian influenza viruses (HPAIVs) isolated in Hokkaido, northern Japan, during the winter of 2024–2025. Ninety-eight HPAIVs were isolated from feces of waterfowl, tracheal swabs from dead wild birds, or lung homogenates from dead chickens. Phylogenetic analysis of the hemagglutinin (HA) gene from 47 representative isolates revealed that all sequences belonged to the G2d subgroup of clade 2.3.4.4b H5HA, which has been the dominant lineage in Hokkaido since the winter of 2021–2022. These isolates were further divided into three major groups within the subgroup. The HPAIVs isolated in the Republic of Korea, China, and North America were genetically closely related to the Hokkaido isolates, whereas no HPAIVs genetically related to European strains or those detected in North American cattle were identified. Furthermore, HPAIVs isolated from seabirds were genetically closely related to those found in dead marine mammals along the eastern coast of Hokkaido in the spring of 2025. No apparent antigenic differences were observed between the HPAIVs isolated in this study and those from previous seasons. These findings highlight the wide distribution of HPAIVs in Hokkaido, particularly from Asian and North American lineages, and underscore the importance of continuous surveillance.

## 1. Introduction

Outbreaks of high pathogenicity avian influenza (HPAI) have reached a global level, with several HPAI virus (HPAIV) infections reported in both poultry and wild birds. Since 2021, HPAIVs, primarily clade 2.3.4.4b H5, have spread from Eurasia to North America, Latin America, and even Antarctica via migratory waterfowl [1,2,3,4]. As the virus has spread, HPAI outbreaks in poultry farms have reached unprecedented levels, with 2466 outbreaks reported in Europe during 2022–2023 and 285 in the USA between February and September 2022 [5,6]. For the first time, HPAI outbreaks were also confirmed in several Latin American countries, including Colombia, Venezuela, Peru, Ecuador, Bolivia, Chile, Argentina, Uruguay, Paraguay, and Brazil [6]. The widespread occurrence of HPAI outbreaks poses a serious threat to the poultry industry and the sustainable supply of chicken products. Similar to other regions, Japan has experienced alarming numbers of HPAIV infections in both poultry and wild birds since 2021. During the winter of 2021–2022, 25 and 107 HPAIV infection cases were reported in poultry and wild birds, respectively; these numbers increased to 84 and 242 in the winter of 2022–2023 [7]. A comparable surge in reported HPAIV infections was also observed in the Republic of Korea [8], suggesting a high regional density of HPAIV. The elevated number of HPAIV infections in Japanese wild birds continued in the winter of 2023–2024. Hokkaido, situated in northern Japan, is a key stopover site for migratory birds, first in autumn when birds arrive from the north, and again in spring when they depart northward. This makes Hokkaido a likely entry point for several contagious viruses and a potential indicator of HPAIV introduction to southern Japan and other parts of the Far East.

The high concentration and wide geographic spread of HPAIVs has led to spillover infections in other avian species where HPAIV infection had previously been rare, including Accipitriformes and Charadriiformes [6]. In Europe, several seagulls were infected with H5 HPAIV beginning in the summer of 2022. Genetic analysis indicated that the EA-2022-BB genotype emerged through genetic reassortment between H5 HPAIV and H13 low pathogenic avian influenza viruses (LPAIVs), which are typically found in seagull populations. This virus caused mass mortality events among infected birds in northern Europe in the summer of 2022, in southern Europe in the autumn of 2022, and again in northern Europe during the winter of 2022–2023 [9]. During the spread of this genotype in Europe, several seabird populations experienced serious declines with mass mortality, particularly in colonies of gulls and terns across several countries [10]. The adaptation of the EA-2022-BB genotype to the family Laridae is attributed to PA, NP, and NS genes derived from H13 LPAIVs. Since this genotype has not been detected in Anseriformes, it is unlikely to be transmitted by migratory waterfowl or to be detected in areas far from the epicenter of this genotype circulation. However, introduction of pathogens with unique characteristics, particularly those with increased risk, should remain a key focus of pathogen surveillance programs. Indeed, H5N8 HPAIVs isolated in Japan in the early winter of 2020–2021 were genetically close to the ones isolated in Europe in the winter of 2019–2020, indicating that HPAIVs can be transported over long distances by migratory birds within a year [11]. The widespread circulation of H5 HPAIVs has also resulted in spillover into wild mammals that share habitats with HPAIV-infected avian species, particularly species within the orders Carnivora and Cetartiodactyla [12]. Notably, a large number of mammalian infections have raised concerns about possible mammal-to-mammal transmission, particularly in mink, marine mammals, and dairy cattle [13,14,15]. High HPAIV titers in cattle milk may represent a potential zoonotic risk to humans [16]. HPAIV infection in dairy cattle was first reported in the United States in March 2024, with symptoms including low feed intake, mild respiratory signs, diarrhea, and discolored milk [14]. Initially, these cases were linked to genotype B3.13 of H5N1 HPAIV in 2024, but a different genotype (D1.1) of H5N1 HPAIVs was subsequently detected in infected dairy cattle in the United States [17]. Considering a report of H5 HPAIV detected in the Republic of Korea that originated from North America, HPAIVs with the potential to infect new hosts could be introduced into the Far East [18]. Within the Far East, interspecies transmission of HPAIV to mammals, including foxes, racoon dogs (tanuki), cats, and marine mammals, has already been reported [19,20,21,22]. In particular, HPAIV infection in marine mammals could have substantial ecological consequences. HPAIVs were detected in carcasses of northern fur seals (*Callorhinus ursinus*) and Steller sea lions (*Eumetopias jubatus*) found on Tyuleniy Island in the summer of 2023. These isolates were genetically similar to viruses detected in Hokkaido [21,22].

In the winter of 2024–2025, 51 and 227 cases of HPAIV infection were reported in poultry farms and wild birds, respectively, of which 2 and 74 cases occurred in Hokkaido [7,23]. Considering geographic proximity and epidemiological connectivity, northern Japan, especially Hokkaido, should be regarded as one of the “preferential” areas where HPAIVs with potential risks tend to converge. In this study, HPAIVs isolated through active surveillance of migratory waterfowl and passive surveillance of dead wild birds and poultry were genetically and antigenically investigated. The results demonstrated that, in the Far East, HPAIVs were distributed over a broad area, including the United States (Alaska) and China. However, virus transmission routes to northern and southern Japan may differ. A better understanding of the unique epidemiological features of HPAIVs in Hokkaido is essential for elucidating intercontinental HPAIV dynamics.

## 2. Materials and Methods

### 2.1. Overview of High Pathogenicity Avian Influenza Virus Infection in the Winter of 2024–2025

**Figure 1:**
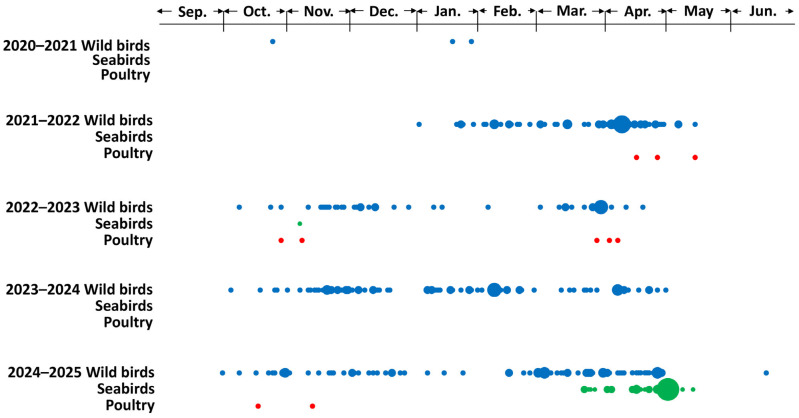
Reports of highly pathogenic avian influenza virus (HPAIV) infections in poultry and wild birds in Hokkaido. Daily reported cases of HPAIV infections from winter 2020–2021 to 2024–2025 are plotted by species: wild birds excluding seabirds (blue), seabirds (green), and poultry (red). The size of each plot is proportional to the number of cases (one case is smallest and nine cases is biggest plots) in each day in category. Data were derived from [7].

An HPAIV was first isolated from fecal samples collected during an active surveillance survey at Notsuke Peninsula (latitude: 43°36′12″ N; longitude: 145°17′35″ E) in eastern Hokkaido on 8 October 2024. Subsequently, HPAIVs were continuously isolated from the carcasses of dead wild birds, including raptors, waterfowl, crows, and seabirds, between 16 October 2024 and 17 June 2025. Regarding HPAI outbreaks in poultry farms in Hokkaido, two outbreaks were reported, on 17 October and 12 November 2024. In the winter of 2024–2025, the first wave of HPAIV infections in wild birds in Hokkaido occurred between October and December 2024, and the second wave occurred between February and May 2025 (Figure 1). A two-wave seasonal pattern of HPAIV infection in wild birds has been observed during each of the past three seasons, although the timing between waves varied across years.

### 2.2. Sample Collection

In total, 101 fecal samples from wild waterfowl were collected at Notsuke Peninsula. These fecal samples were mixed with a virus transport medium comprising minimum essential medium (Shimazu Diagnostics, Tokyo, Japan) supplemented with 10 mg/mL streptomycin (Meiji Seika Pharma, Tokyo, Japan), 10,000 U/mL penicillin G (Meiji Seika Pharma, Tokyo, Japan), 250 U/mL nystatin (Sigma-Aldrich, St. Louis, MO, USA), 0.3 mg/mL gentamicin (MSD, Tokyo, Japan), and 0.5% bovine serum albumin fraction V (Roche, Basel, Switzerland).

In the present study, three types of passive surveillance of HPAIV infections in dead birds found in Hokkaido were adopted. As a sentinel survey conducted in an urban garden in Sapporo, Hokkaido, tracheal swabs were collected from all the 61 crow carcasses found there to perform the definitive diagnosis of HPAIV infection in Hokkaido University. Detailed species of crows were identified morphologically except one crow carcass without the head. For the monitoring of circulating HPAIV, tracheal swabs of other dead wild birds found throughout Hokkaido were provided on an ad hoc basis from the National Institute of Environmental Studies (NIES) to isolate HPAIV following the implementation of the definitive diagnosis of HPAIV infection in NIES. As part of pathological investigations, lung homogenates from dead chickens suspected of HPAIV infection in two poultry farms were provided by the Hokkaido Prefectural Government, though the definitive investigation of these cases of HPAIV infection was conducted by the national or prefectural government.

### 2.3. Virus Isolation and Confirmation of Pathogenicity of Virus Isolates

Fecal, tracheal swab, and lung homogenate samples were inoculated into three 10-days-old embryonated chicken eggs per sample for virus isolation. After 48 h incubation at 35 °C, virus replication in the eggs was confirmed by the hemagglutination assay (HA) using the allantoic fluid of the eggs. Because AIVs isolated from fecal samples from migratory wild birds and tracheal swab samples from crows had not yet been diagnosed as H5 subtype or HPAIV, the viral subtype and pathogenicity were determined by quantitative reverse transcription-polymerase chain reaction (RT-qPCR) and direct sequencing of the HA cleavage site. Viral RNAs were extracted from the allantoic fluid of eggs using the QIAamp Viral RNA Mini Kit (Qiagen, Hilden, Germany). The presence of the H5 HA gene was investigated by RT-qPCR using AgPath-ID One-Step RT-PCR Reagents (Thermo Fisher Scientific, Waltham, MA, USA) on a StepOnePlus Real-Time PCR System (Thermo Fisher Scientific), as described by Heine et al. [24]. After confirmation of the HA subtype, the isolates were subjected to PCR using region-specific primers [25], followed by direct sequencing to confirm the presence of multiple basic amino acid residues at the HA cleavage site, a molecular marker for high pathogenicity in avian influenza viruses (AIVs).

### 2.4. DNA Barcoding

To determine the host species of the AIV-positive fecal samples, DNA was extracted using the NucleoSpin^®^ DNA Stool Kit (Takara bio, Shiga, Japan). A 749-base pair fragment near the 5′-terminus of the cytochrome *c* oxidase (COI) gene was amplified using the primers described previously [26]. The PCR product was sequenced and compared to the sequence data of the COI gene in the Barcode of Life Data Systems (http://www.barcodinglife.com (accessed on 14 July 2025)) for species identification.

### 2.5. Genetic Analysis

For representative virus isolates selected based on spatial and temporal distribution and host species, the whole genome was sequenced to determine the NA subtype of the isolate and perform genetic analysis. Next-generation sequencing was performed using the Flongle platform (Oxford Nanopore Technologies) with primers designed to amplify all eight gene segments of HPAIV isolates, following the method described by Ip et al. [27]. Oxford Nanopore sequencing libraries were prepared using the NEB Ultra II End Repair/dA-Tailing Module (New England Biolabs, Ipswich, MA, USA) and sequenced on Flongle using the Ligation Sequencing Kit V14 (Oxford Nanopore Technologies, Oxford, UK). The obtained reads were mapped and assembled using FluGAS version 2 (World Fusion, Tokyo, Japan).

Nucleotide sequences of the isolates were phylogenetically analyzed based on the maximum likelihood method using the best-fit general time-reversible model of nucleotide substitution with gamma-distributed rate variation among sites (with four rate categories, Γ), according to the Tamura–Nei model [28]. Bootstrap analysis with 1000 replicates was conducted to construct the phylogenetic trees in MEGA 7 with default parameters [29]. Sequence data of the genes were compared to reference nucleotide sequences of representative H5Nx viruses (with different NA subtypes) in clade 2.3.4.4, downloaded from the Global Initiative on Sharing All Influenza Data (GISAID; https://gisaid.org/ accessed on 14 July 2025). Internal gene segments were also analyzed to identify potential reassortment by comparing their phylogenetic patterns with those of the HA gene.

To examine the evolutionary relationships and spatial spread of H5 HPAIV isolates in Hokkaido, a time-calibrated Maximum Clade Credibility (MCC) tree of the H5 HA gene was reconstructed using BEAST v2.7.6 [30] with the HKY+G4 nucleotide substitution model [31] and a relaxed lognormal molecular clock [32]. Analyses were run for 10 million steps, sampling every 1000 steps, and the first 10% of samples were discarded as burn-in. Convergence was assessed in Tracer v1.7, confirming that all effective sample size values exceeded 200 [33]. The MCC tree was summarized with TreeAnnotator v1.10.4 [34] and visualized in R v2.4.2 using the ggtree package [35]. Geographic states were assigned to isolates based on their sampling locations, categorized into five regions: Japan (subdivided into Hokkaido and the main island), Northeast Asia (China and South Korea), Russia, the Americas (North and South America), and Southeast Asia (Vietnam). These states were mapped onto tips and inferred for ancestral nodes, facilitating the reconstruction of spatial diffusion patterns and the estimation of the most likely geographic origins of internal nodes.

### 2.6. Antigenic Analysis

The antigenicity of HPAIVs isolated in the winter of 2024–2025 was compared to that of HPAIVs isolated in the past season in Japan using a cross-hemagglutination inhibition (HI) test, as described in a previous study [36]. In total, 29 H5 AIV strains, including 6 isolates from this study and 14 hyperimmune sera, were used for the cross-HI test (Supplementary Table S1). Antigenic cartography was generated based on the cross-HI test results using the Racmac package in the R program version 4.3.0 [37]. HI titers were converted into antigenic distances by log_2_ transformation, where a two-fold difference in titer corresponds to one unit of antigenic distance. The x–y coordinates of antigens and antisera were estimated through multidimensional scaling to minimize the difference between observed and mapped distances, enabling a visual representation of antigenic relationships.

In order to assess the significance of the antigenicity difference between two distinct clades, the x–y coordinates of antigens and antisera in each clade were calculated and the centroid of the coordinates was set as the antigenic center coordinate. The antigenic variance of the antigens and antisera in the clade was defined as the variance of the distances between the antigenic center coordinate to each coordinate onto the line through two antigenic center coordinates from each antigen/antisera coordinate. If the distance between two antigenic center coordinates is significant given both of the antigenic variance of the antigens and antisera, the antigenicity of these two clades was regarded as significantly different. Significance was confirmed with *p* < 0.05, and this statistical analysis was applied to the set of the clade which had more than three antigens or antisera.

## 3. Results

### 3.1. Virus Isolation

In the winter of 2024–2025, 1 of 101 fecal samples from wild waterfowl and 47 from crow carcasses tested positive for AIV infection. All isolates were confirmed as HPAIV subtyped as H5. According to the COI gene database, the mitochondrial DNA sequence extracted from the fecal sample (No. 76) exhibited 100% homology with the COI gene of the Eurasian wigeon (Mareca penelope). The HPAIV isolate from this sample was designated as A/Eurasian wigeon/Hokkaido/Q76/2024 (H5N1: Ew/Q76/24) and subtyped through genetic analysis. Host birds of all HPAIV-positive crow carcasses were identified morphologically, and isolates were appropriately designated. In addition, 44 and 6 HPAIVs were successfully isolated from swab samples of dead wild birds and lung homogenates from dead chickens in H5 HPAI-affected poultry farms, respectively. The whole sequences of all eight gene segments from 47 HPAIV isolates were submitted to the GISAID database (Table 1).

### 3.2. Genetic Analysis

**Figure 2:**
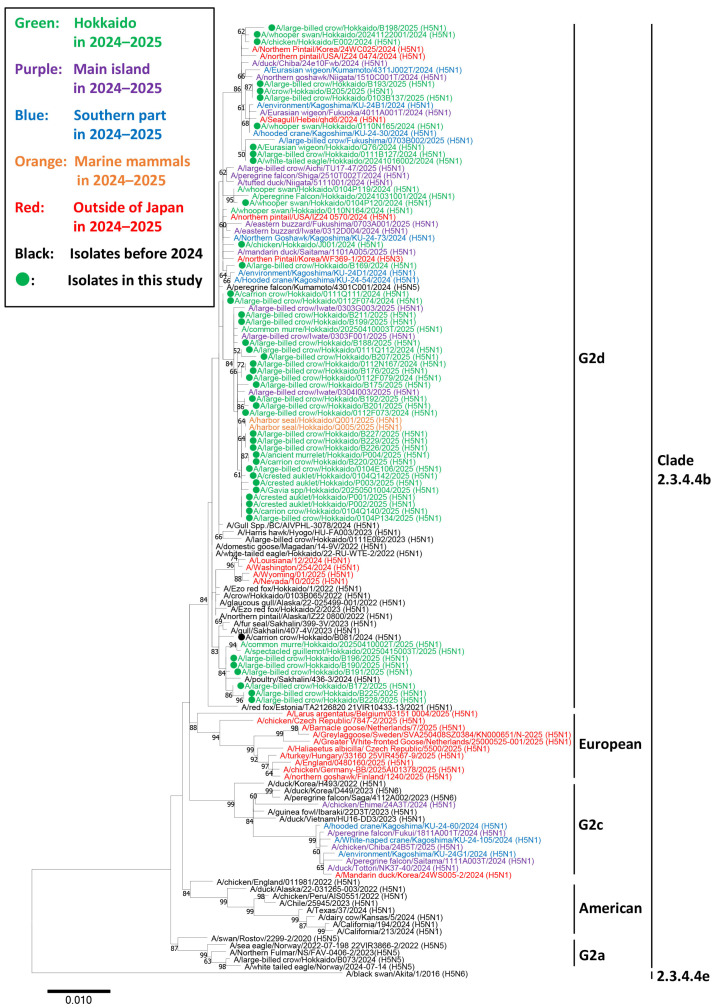
Phylogenetic analysis of the hemagglutinin gene segment of highly pathogenic avian influenza viruses (HPAIVs) isolated in Hokkaido, Japan in the winter of 2024–2025. Hemagglutinin (HA) gene sequences of the viruses isolated in this study and H5 HPAIVs obtained from the Global Initiative on Sharing Avian Influenza Data were used for constructing a phylogenetic tree. The green-colored strains indicate H5 HPAIVs isolated from avian species in Hokkaido in the winter of 2024–2025. The purple- and blue-colored strains indicate H5 HPAIVs isolated from the main island and southern Japan (Kagoshima), respectively, during the same period. The red-colored strains indicate H5 HPAIVs isolated outside Japan, including European countries, the United States, Canada, the Republic of Korea, and China, during the same period. The orange-colored strains indicate H5 HPAIVs isolated from marine mammals in Hokkaido during the same period. Strains isolated in this study are marked with filled circles. Bootstrap values >60% are indicated at the corresponding nodes.

In the phylogenetic analysis of the H5 HA gene, all the 47 strains used for genetic analysis were subtyped as H5N1 and categorized in clade 2.3.4.4b and classified into the G2d subgroup. In contrast, most HPAIVs isolated in other regions of Japan clustered in a different subgroup (G2c) (Figure 2). In addition, other HPAIVs isolated in Hokkaido during the same season also belonged to the G2d subgroup. None of the European genotypes prevalent in the winter of 2024–2025, including EA-2024-DI.1 (A/Greater White-fronted Goose/Netherlands/25000525-001/2025 (H5N1)), -DI.2 (A/chicken/Germany-BB/2025AI01378/2025 (H5N1)), -BB (A/Larus argentatus/Belgium/03151_0004/2025 (H5N1)), -DA (A/chicken/Czech Republic/7847-2/2025 (H5N1)), and -EF (A/Barnacle Goose/Netherlands/7/2025 (H5N1)), were detected in Hokkaido. Similarly, none of the genotypes associated with cattle infections in North America, including genotypes B3.13 (A/California/194/2024 (H5N1)) and D1.1 (A/Nevada/10/2025 (H5N1)), were isolated in Hokkaido (Figure 2).

Within the G2d subgroup, three major clusters were identified based on the phylogenetic tree structure. Cluster 1 included Ew/Q76/24 and was genetically related to HPAIVs isolated in the United States (A/northern pintail/USA/IZ24_0474/2024 (H5N1)), China (A/seagull/Hebei/qhd6/2024 (H5N1)), and the Republic of Korea (A/northern pintail/Korea/24Wc025/2024 (H5N1)). Cluster 2 contained several HPAIV isolates from Sapporo or Sakhalin, which were genetically close to an HPAIV isolated in Sakhalin during the previous winter (A/poultry/Sakhalin/436-3/2024 (H5N1)). This cluster also included two HPAIV isolates from Alaska (A/northern pintail/Alaska/IZ22_0800/2022 (H5N1) and A/northern pintail/Alaska/22-025499-001/2022 (H5N1)), which were located closely in the phylogenetic tree. Cluster 3 comprised isolates from crows, seabirds, and marine mammals in Hokkaido. These viruses were isolated from across Hokkaido between November 2024 and April 2025, suggesting that this group should be widely prevalent throughout the region during the winter season.

**Figure 3:**
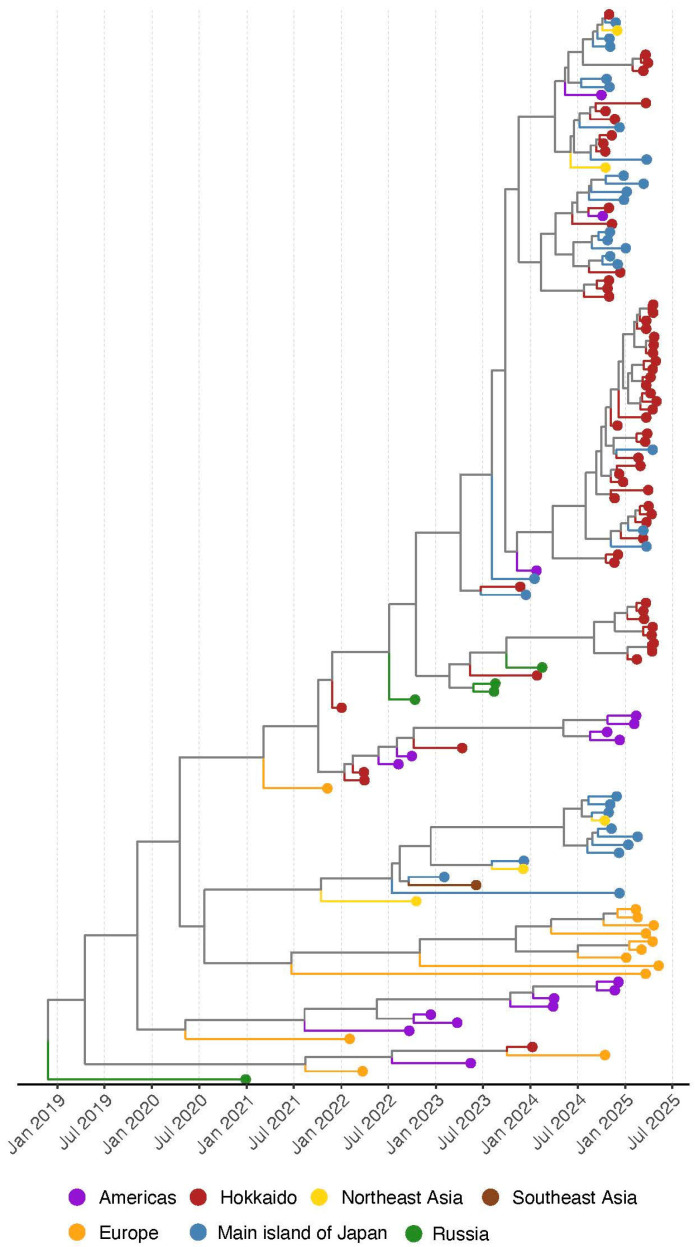
Maximum clade credibility (MCC) tree reconstructed from the hemagglutinin gene of H5Nx high pathogenicity avian influenza viruses (clade 2.3.4.4b) collected in Japan (Hokkaido and the main islands) from 2021 to 2025. To provide geographic context, isolates from Northeast Asia (China and South Korea), Russia, the Americas (North and South America), and Southeast Asia (Vietnam) collected during the same period are also included. Geographic origins are indicated by different colors on the external nodes of the MCC. Detailed strain information is in Supplementary Figure S2.

In the time-calibrated MCC tree of the H5 HA, it was indicated that all of the HPAIV isolates in the winter of 2024–2025 were genetically distinguished from ancestral strains within one year (after July 2024) (Figure 3). Two strains isolated in the Americas (A/northern pintail/USA/IZ24_0474/2024 (H5N1) and (A/northern pintail/USA/IZ24_0570/2024 (H5N1) have the same ancestral strains with Japanese isolates and were genetically divided in the summer 2024 (Supplementary Figure S2). On the other hand, two major clusters in the MCC trees, which were the same as Cluster 1 and 3 in the phylogenetic tree of the HA, were composed of the isolates in Japan (Hokkaido and the main island of Japan). These results imply that HPAIV isolates in the winter of 2024–2025 in Japan have the same ancestral strains with isolates in different regions and were brought to Hokkaido through different pathways.

### 3.3. Antigenic Analysis

**Figure 4:**
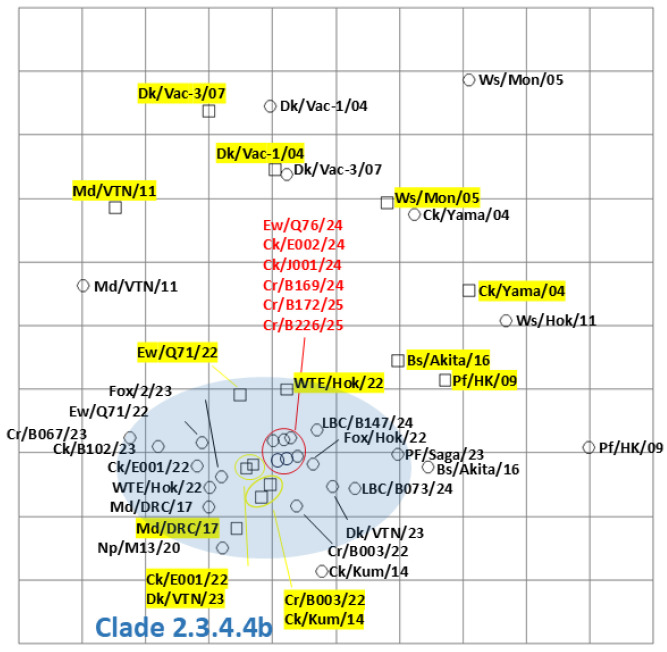
Antigenic map of H5 highly pathogenic avian influenza viruses (HPAIVs) based on cross-hemagglutination inhibition (HI) tests. Antigenic relationships among clade 2.3.4.4 H5 HPAIVs, including seven viruses isolated in Hokkaido in the winter of 2024–2025, are visualized using antigenic cartography, as indicated in Supplementary Table S1. In the antigenic map, horizontal and vertical axes indicate antigenic distances of spacing between the grid lines, where one grid corresponds to a two-fold HI titer difference. The HPAIVs isolated in this study are indicated in red. The antisera are highlighted in yellow. The blue-highlighted area indicates viruses and the sera in clade 2.3.4.4b H5 HPAIVs. Detailed strain information is in Supplementary Table S1.

Among the isolates in this study, six viruses, Ew/Q76/24, A/chicken/Hokkaido/E002/2024 (H5N1: Ck/E002/24), A/chicken/Hokkaido/J001/2024 (H5N1: Ck/J001/24), A/large-billed crow/Hokkaido/B169/2024 (H5N1: Cr/B169/24), A/large-billed crow/Hokkaido/B172/2025 (H5N1: Cr/B172/25), and A/large-billed crow/Hokkaido/B226/2025 (H5N1: Cr/B226/25), were selected for antigenic analysis by the cross-HI test. Consistent with other clade 2.3.4.4b H5 HPAIVs, the six HPAIVs isolated in the winter of 2024–2025 exhibited homologous reactions with antisera against A/large-billed crow/Hokkaido/B003/2022 (H5N2), A/chicken/Hokkaido/E001/2022 (H5N1), A/Eurasian wigeon/Hokkaido/Q71/2022 (H5N1), and A/white-tailed eagle/Hokkaido/22-RU-WTE-2/2022 (H5N1) (Supplementary Table S1). Reactivity patterns were similar among the isolates and consistent with previously characterized clade 2.3.4.4b H5 HPAIVs. Antigenic cartography demonstrated that the antigenicity of HPAIVs isolated in the winter of 2024–2025 was almost identical and similar to that of clade 2.3.4.4b H5 HPAIVs previously isolated (Figure 4). Antigenicity of clade 2.3.4.4b was significantly different from that of Classic (A/duck/Hokkaido/Vac-1/2004 (H5N1) and A/duck/Hokkaido/Vac-3/2007 (H5N1)), and that of clade 2.5 (A/whooper swan/Mongolia/3/2005 (H5N1) and A/chicken/Yamaguchi/7/2004 (H5N1)).

## 4. Discussion

From October 2024 to June 2025, several HPAIV infection events were recorded in Hokkaido, primarily affecting wild birds, along with two outbreaks in poultry farms. Genetic analysis indicated that viruses responsible for the poultry outbreaks were closely related to HPAIVs prevalent in wild birds during the same period, suggesting that these HPAI outbreaks likely resulted from spillover transmission from wild birds. Hokkaido has consistently accounted for a high proportion of HPAIV cases reported in Japan during recent winter seasons: 39 of 242 cases in 2022–2023, 70 of 156 in 2023–2024, and 74 of 227 in 2024–2025 [7]. This can be attributed not only to the large area and wildlife density of Hokkaido but also to its geographic positioning. Located at the northernmost edge of Japan, Hokkaido serves as both the first stopover site for migratory waterfowl in autumn and the final departure point in spring. As a result, this region experiences a prolonged window of potential HPAIV exposure compared to other prefectures in Japan. This extended season exposure increases the likelihood of repeated introductions of diverse HPAIVs into Hokkaido every winter.

In this study, we demonstrated that HPAIV isolates in Hokkaido exhibited genetic diversity within the G2d subgroup. Based on the spatial distribution of virus isolates, the genetic group including Ew/Q76/24 was widely disseminated across regions. Detection of viruses in this group during the early phase of the winter season (between October and December 2024) implies that these viruses might have originated from a common source to both the western part of North America and the Far East through long-distance bird migration, although the precise origin of the viruses remains unidentified [38]. The previous report of H5N1 HPAIV originating from the western part of North American breeding grounds, being isolated in the Republic of Korea in the autumn of 2022, supports the feasibility of inter-Beringian transmission events and the possible virus pools in Siberia-Alaska [18]. In contrast, the remaining two genetic groups in G2d were regionally confined. One group was shared between Sakhalin (the eastern part of Russia) and Sapporo (the central part of Hokkaido), where recurrent crow die-offs have been reported in a public garden in the central part of Sapporo every winter–spring since 2022 [39]. The concentration of virus detections in Sapporo may reflect sampling bias, rather than distinct local epidemiological factor(s). However, the continuous invasion of the HPAIVs in the same subgroups in three successive winter seasons without virus sustainment in summer seasons implies that unique and inherent bird migrations should be likely to be responsible for intra-regional HPAIV spread. Considering the elusive nature of wild birds, particularly migratory waterfowl and resident birds, detection of wild bird carcasses is quite low in the absence of active surveillance. The other group comprised isolates detected through active surveillance of crows in Sapporo and of dead seabirds and harbor seals (*Phoca vitulina*) in eastern Hokkaido, as reported on the website of the competent authority [7]. Interestingly, HPAIVs isolated from seabirds, such as the crested auklet (*Aethia cristatella*), ancient murrelet (*Synthliboramphus antiquus*), and *Gavia* spp., since late March 2025, were genetically very close to viruses isolated from crows in the same region and in Sapporo in mid-April 2025. However, they were distinct from the HPAIVs previously isolated from northern fur seals on Tyuleniy Island in 2023 [22]. Although HPAIV infections in seabirds in Hokkaido were rarely reported before the 2023–2024 winter, several cases were reported in mid-March in the winter of 2024–2025. The viruses isolated from these seabirds were also genetically very close to those isolated from harbor seals from the same regions, although detailed entomological and epidemiological reports regarding the die-offs of the seabirds and marine mammals around this area in the spring of 2025 are lacking [7]. It is plausible that HPAIVs detected in both eastern Hokkaido and the crow population in Sapporo were newly introduced at the end of winter, probably by northward migration of birds, rather than through sustained local circulation following early-season virus introductions. Thereafter, these viruses may have persisted in these regions, and in eastern Hokkaido, overlapping habitats of resident birds and seabirds may have facilitated die-offs in both avian and mammalian species. Further investigation of these events is crucial to better understand the epidemiology and ecology of HPAIVs and to assess the potential risks they pose to resident animals in this area. Overall, these data highlight that H5 HPAIVs circulating in the Far East exhibit genetic diversity, distinct host associations, and epidemiological patterns. However, the ability to cause adverse effects in mammals may not be limited to only certain genetic groups.

In this study, we demonstrated that HPAIV isolates in Hokkaido exhibited genetic diversity in the G2d subgroup; however, no significant antigenic variation was observed among them. A previous study reported an antigenic difference between two HPAIVs belonging to the same G2d subgroup, despite their similar pathogenicity in chickens [40]. This antigenic gap was attributed to amino acid substitutions at positions 189 and 193 of the HA protein, as identified by Kaverin et al. [41]. Among the six HPAIVs isolated in the winter of 2024–2025 and analyzed by cross-HI testing in this study, three amino acid substitutions were confirmed in the HA protein of the six strains; Ck/J001/24: F233Y, Cr/B172/25: N313S, and Cr/B226/25: K329R. However, none of these substitutions corresponded to the key amino acid residues influencing the antigenicity of clade 2.3.4.4 H5 HPAIV, which was reported by Zhang et al. [42]. Considering the genetic diversity and broad variation of H5 HPAIV, predicting HPAIV characteristics based on amino acid sequence is informative. Therefore, continued surveillance of genetic and antigenic properties is essential.

Several HPAIVs with potentially different characteristics have been isolated worldwide. However, there remains a risk of their introduction through bird migrations, because cross-continental HPAIV spread along overlapping flyways of migratory birds has already been documented [43]. The migratory route through Kuril Islands-Kamchatka Peninsula to eastern Hokkaido could be a reasonable flyway introducing the viruses from the western part of North America. In addition, HPAIV movements within Hokkaido could also be overlapped with the migration route within Hokkaido [43]. In the Far East, HPAIVs originating from Europe, Asia, and North America are believed to converge, and Hokkaido, in particular, may serve as a “seasonal hub” for these viruses as it was indicated that HPAIV isolates in several regions may have the same ancestral strains. Though there are no official records, it is said that migratory birds usually come from the north to Hokkaido Island between the middle of September and the middle of October, and come from the south to Hokkaido Island between the beginning of April to the beginning of May, which is consistent with HPAIV infection cases reported in Hokkaido (Figure 1). Given these epidemiological findings through bird migration, a variety of genetic patterns of the HA genes in H5 HPAIV isolates was reasonable although the potential challenges and limitations in the present study should remain. One of the biggest concerns potentially affecting the output of the analyses was that the available dataset might be biased. It is natural that the output of the genetic data heavily depend on the amount of the sequence data and its geographical distributions. A proper dataset is required to obtain reasonable and valid conclusions, especially in the genetic analysis. The results of this study underscore the importance of continuous monitoring of HPAIVs to characterize their evolution and support timely implementation of control measures should high-risk strains be detected.

## Figures and Tables

**Table 1:** Highly pathogenic avian influenza viruses sequenced and used in the present study.

ID	Virus	Subtype	Date of Samples Found	City Name Sampled	Accession Number
1	A/Eurasian wigeon/Hokkaido/Q76/2024	H5N1	8 October 2024	Betsukai	EPI_ISL_19476809
2	A/white-tailed eagle/Hokkaido/20241016002/2024	H5N1	16 October 2024	Shari	EPI_ISL_19504792
3	A/chicken/Hokkaido/E002/2024	H5N1	17 October 2024	Atsuma	EPI_ISL_19504793
4	A/whooper swan/Hokkaido/0104P120/2024	H5N1	30 October 2024	Kushiro	EPI_ISL_19574220
5	A/whooper swan/Hokkaido/0110N164/2024	H5N1	30 October 2024	Ikeda	EPI_ISL_19574223
6	A/whooper swan/Hokkaido/0110N165/2024	H5N1	31 October 2024	Honbetsu	EPI_ISL_19574224
7	A/large-billed crow/Hokkaido/0111B127/2024	H5N1	10 November 2024	Sapporo	EPI_ISL_19574222
8	A/chicken/Hokkaido/J001/2024	H5N1	11 November 2024	Asahikawa	EPI_ISL_19574217
9	A/carrion crow/Hokkaido/0111Q111/2024	H5N1	20 November 2024	Nemuro	EPI_ISL_19666682
10	A/large-billed crow/Hokkaido/0111Q112/2024	H5N1	21 November 2024	Nemuro	EPI_ISL_19666681
11	A/whooper swan/Hokkaido/20241122001/2024	H5N1	22 November 2024	Abashiri	EPI_ISL_19666579
12	A/large-billed crow/Hokkaido/0112F073/2024	H5N1	2 December 2024	Erimo	EPI_ISL_19666680
13	A/large-billed crow/Hokkaido/0112F074/2024	H5N1	4 December 2024	Erimo	EPI_ISL_19666679
14	A/large-billed crow/Hokkaido/0112N167/2024	H5N1	9 December 2024	Hiroo	EPI_ISL_19666667
15	A/large-billed crow/Hokkaido/B169/2024	H5N1	13 December 2024	Sapporo	EPI_ISL_19666665
16	A/large-billed crow/Hokkaido/0112F079/2024	H5N1	24 December 2024	Erimo	EPI_ISL_19835913
17	A/large-billed crow/Hokkaido/B172/2025	H5N1	14 February 2025	Sapporo	EPI_ISL_19750619
18	A/large-billed crow/Hokkaido/B175/2025	H5N1	20 February 2025	Sapporo	EPI_ISL_19906326
19	A/large-billed crow/Hokkaido/B176/2025	H5N1	28 February 2025	Sapporo	EPI_ISL_19888043
20	A/large-billed crow/Hokkaido/B188/2025	H5N1	10 March 2025	Sapporo	EPI_ISL_19906342
21	A/large-billed crow/Hokkaido/0103B137/2025	H5N1	11 March 2025	Sapporo	EPI_ISL_19835914
22	A/large-billed crow/Hokkaido/B190/2025	H5N1	11 March 2025	Sapporo	EPI_ISL_20127437
23	A/large-billed crow/Hokkaido/B191/2025	H5N1	11 March 2025	Sapporo	EPI_ISL_19906344
24	A/large-billed crow/Hokkaido/B192/2025	H5N1	18 March 2025	Sapporo	EPI_ISL_19888044
25	A/large-billed crow/Hokkaido/B193/2025	H5N1	20 March 2025	Sapporo	EPI_ISL_19906347
26	A/large-billed crow/Hokkaido/B196/2025	H5N1	21 March 2025	Sapporo	EPI_ISL_19906348
27	A/large-billed crow/Hokkaido/B198/2025	H5N1	21 March 2025	Sapporo	EPI_ISL_19906349
28	A/crested auklet/Hokkaido/P001/2025	H5N1	22 March 2025	Kushiro	EPI_ISL_19855235
29	A/crested auklet/Hokkaido/P002/2025	H5N1	22 March 2025	Kushiro	EPI_ISL_19855236
30	A/crested auklet/Hokkaido/P003/2025	H5N1	22 March 2025	Kushiro	EPI_ISL_19855237
31	A/ancient murrelet/Hokkaido/P004/2025	H5N1	22 March 2025	Kushiro	EPI_ISL_19855238
32	A/large-billed crow/Hokkaido/B199/2025	H5N1	23 March 2025	Sapporo	EPI_ISL_19906350
33	A/large-billed crow/Hokkaido/B201/2025	H5N1	26 March 2025	Sapporo	EPI_ISL_19888057
34	A/crow/Hokkaido/B205/2025	H5N1	29 March 2025	Sapporo	EPI_ISL_20127467
35	A/large-billed crow/Hokkaido/B207/2025	H5N1	30 March 2025	Sapporo	EPI_ISL_20127492
36	A/large-billed crow/Hokkaido/B211/2025	H5N1	31 March 2025	Sapporo	EPI_ISL_20127549
37	A/large-billed crow/Hokkaido/0104P134/2025	H5N1	8 April 2025	Kushiro	EPI_ISL_19908842
38	A/large-billed crow/Hokkaido/B220/2025	H5N1	8 April 2025	Sapporo	EPI_ISL_20127556
39	A/large-billed crow/Hokkaido/B225/2025	H5N1	8 April 2025	Sapporo	EPI_ISL_19906351
40	A/carrion crow/Hokkaido/0104Q140/2025	H5N1	16 April 2025	Nemuro	EPI_ISL_19908843
41	A/crested auklet/Hokkaido/0104Q142/2025	H5N1	16 April 2025	Nemuro	EPI_ISL_19908844
42	A/large-billed crow/Hokkaido/B226/2025	H5N1	17 April 2025	Sapporo	EPI_ISL_19888058
43	A/large-billed crow/Hokkaido/B227/2025	H5N1	20 April 2025	Sapporo	EPI_ISL_19888059
44	A/large-billed crow/Hokkaido/B228/2025	H5N1	20 April 2025	Sapporo	EPI_ISL_20127601
45	A/large-billed crow/Hokkaido/B229/2025	H5N1	22 April 2025	Sapporo	EPI_ISL_18830860
46	A/large-billed crow/Hokkaido/0104E106/2025	H5N1	28 April 2025	Tomakomai	EPI_ISL_19908915
47	A/Gavia spp/Hokkaido/20250501004/2025	H5N1	1 May 2025	Betsukai	EPI_ISL_19908916

Geographic information of cities are described in Supplementary Figure S1.

## Data Availability

The data presented in this study are openly available in the Global Initiative on Sharing All Influenza Data. All the accession numbers of the virus sequence data are described in Table 1.

## Supplementary Materials

The following supporting information can be downloaded at: https://www.mdpi.com/article/10.3390/pathogens14090951/s1, Supplementary Table S1: Cross-hemagglutination inhibition tests of H5 high pathogenicity avian influenza viruses isolated in Hokkaido in winter 2024–2025. Supplemental Figure S1: location of HPAIV positive bird samples found in Hokkaido in winter 2024–2025 season. Supplemental Figure S2. Maximum clade credibility (MCC) tree reconstructed from the hemagglutinin gene of H5Nx high pathogenicity avian influenza viruses (clade 2.3.4.4b) collected in Japan (Hokkaido and the main islands) from 2021 to 2025.

## References

[1] Banyard A.C., Bennison A., Byrne A.M.P., Reid S.M., Lynton-Jenkins J.G., Mollett B., De Silva D., Peers-Dent J., Finlayson K., Hall R. (2024). Detection and spread of high pathogenicity avian influenza virus H5N1 in the Antarctic Region. Nat. Commun..

[2] Caliendo V., Lewis N.S., Pohlmann A., Baillie S.R., Banyard A.C., Beer M., Brown I.H., Fouchier R.A.M., Hansen R.D.E., Lameris T.K. (2022). Transatlantic spread of highly pathogenic avian influenza H5N1 by wild birds from Europe to North America in 2021. Sci. Rep..

[3] Jimenez-Bluhm P., Siegers J.Y., Tan S., Sharp B., Freiden P., Johow M., Orozco K., Ruiz S., Baumberger C., Galdames P. (2023). Detection and phylogenetic analysis of highly pathogenic A/H5N1 avian influenza clade 2.3.4.4b virus in Chile, 2022. Emerg. Microbes Infect..

[4] Ruiz-Saenz J., Martinez-Gutierrez M., Pujol F.H. (2023). Multiple introductions of highly pathogenic avian influenza H5N1 clade 2.3.4.4b into South America. Travel. Med. Infect. Dis..

[5] Animal and Plant Health Inspection Service U.S.D.A. Confirmation of Highly Pathogenic Avian Influenza in Commercial and Backyard Flocks. https://www.aphis.usda.gov/livestock-poultry-disease/avian/avian-influenza/hpai-detections/commercial-backyard-flocks.

[6] Adlhoch C., Fusaro A., Gonzales J.L., Kuiken T., Mirinaviciute G., Niqueux E., Staubach C., European Food Safety Authority, European Centre for Disease Prevention and Control, European Union Reference Laboratory for Avian Influenza (2023). Avian influenza overview June–September 2023. EFSA J..

[7] Environment of the Ministry Information Regarding High Pathogenicity Avian Influenza. https://www.env.go.jp/nature/dobutsu/bird_flu/index.html.

[8] Lee S.H., Kwon J.H., Youk S., Lee S.W., Lee D.H., Song C.S. (2025). Epidemiology and pathobiology of H5Nx highly pathogenic avian influenza in South Korea (2003–2024): A comprehensive review. Vet. Q..

[9] Adlhoch C., Fusaro A., Gonzales J.L., Kuiken T., Melidou A., Mirinaviciute G., Niqueux E., European Food Safety Authority, European Centre for Disease Prevention and Control, European Union Reference Laboratory for Avian Influenza (2023). Avian influenza overview April–June 2023. EFSA J..

[10] Fusaro A., Zecchin B., Giussani E., Palumbo E., Aguero-Garcia M., Bachofen C., Balint A., Banihashem F., Banyard A.C., Beerens N. (2024). High pathogenic avian influenza A(H5) viruses of clade 2.3.4.4b in Europe-Why trends of virus evolution are more difficult to predict. Virus Evol..

[11] Isoda N., Twabela A.T., Bazarragchaa E., Ogasawara K., Hayashi H., Wang Z.J., Kobayashi D., Watanabe Y., Saito K., Kida H. (2020). Re-Invasion of H5N8 High Pathogenicity Avian Influenza Virus Clade 2.3.4.4b in Hokkaido, Japan, 2020. Viruses.

[12] Fusaro A., Gonzales J.L., Kuiken T., Mirinaviciute G., Niqueux E., Stahl K., Staubach C., European Food Safety Authority, European Centre for Disease Prevention and Control, European Union Reference Laboratory for Avian Influenza (2024). Avian influenza overview December 2023–March 2024. EFSA J..

[13] Aguero M., Monne I., Sanchez A., Zecchin B., Fusaro A., Ruano M.J., Del Valle Arrojo M., Fernandez-Antonio R., Souto A.M., Tordable P. (2023). Highly pathogenic avian influenza A(H5N1) virus infection in farmed minks, Spain, October 2022. Eurosurveillance.

[14] Caserta L.C., Frye E.A., Butt S.L., Laverack M., Nooruzzaman M., Covaleda L.M., Thompson A.C., Koscielny M.P., Cronk B., Johnson A. (2024). Spillover of highly pathogenic avian influenza H5N1 virus to dairy cattle. Nature.

[15] Leguia M., Garcia-Glaessner A., Munoz-Saavedra B., Juarez D., Barrera P., Calvo-Mac C., Jara J., Silva W., Ploog K., Amaro L. (2023). Highly pathogenic avian influenza A (H5N1) in marine mammals and seabirds in Peru. Nat. Commun..

[16] Spackman E., Jones D.R., McCoig A.M., Colonius T.J., Goraichuk I.V., Suarez D.L. (2024). Characterization of highly pathogenic avian influenza virus in retail dairy products in the US. J. Virol..

[17] Owusu H., Sanad Y.M. (2025). Comprehensive Insights into Highly Pathogenic Avian Influenza H5N1 in Dairy Cattle: Transmission Dynamics, Milk-Borne Risks, Public Health Implications, Biosecurity Recommendations, and One Health Strategies for Outbreak Control. Pathogens.

[18] Kang Y.M., Heo G.B., An S.H., Lee Y.N., Cha R.M., Cho H.K., Sagong M., Kim D.H., Lee E.K., Kang H.M. (2023). Introduction of Multiple Novel High Pathogenicity Avian Influenza (H5N1) Virus of Clade 2.3.4.4b into South Korea in 2022. Transbound. Emerg. Dis..

[19] Hiono T., Kobayashi D., Kobayashi A., Suzuki T., Satake Y., Harada R., Matsuno K., Sashika M., Ban H., Kobayashi M. (2023). Virological, pathological, and glycovirological investigations of an Ezo red fox and a tanuki naturally infected with H5N1 high pathogenicity avian influenza viruses in Hokkaido, Japan. Virology.

[20] Kim I.H., Nam J.H., Kim C.K., Choi Y.J., Lee H., An B.M., Lee N.J., Jeong H., Lee S.Y., Yeo S.G. (2024). Pathogenicity of Highly Pathogenic Avian Influenza A(H5N1) Viruses Isolated from Cats in Mice and Ferrets, South Korea, 2023. Emerg. Infect. Dis..

[21] Panova A.S., Kolosova N.P., Svyatchenko S.V., Goncharova N.I., Danilenko A.V., Boldyrev N.D., Shadrinova K.N., Vasiltsova N.N., Egorova M.L., Onkhonova G.S. (2025). Genetic diversity of A(H5N1) avian influenza viruses isolated from birds and seals in Russia in 2023. Sci. Rep..

[22] Sobolev I., Alekseev A., Sharshov K., Chistyaeva M., Ivanov A., Kurskaya O., Ohlopkova O., Moshkin A., Derko A., Loginova A. (2024). Highly Pathogenic Avian Influenza A(H5N1) Virus Clade 2.3.4.4b Infections in Seals, Russia, 2023. Emerg. Infect. Dis..

[23] Ministry of Agriculture, Forestry and Fisheries Information of Avian Influenza. https://www.maff.go.jp/j/syouan/douei/tori/.

[24] Heine H.G., Foord A.J., Wang J., Valdeter S., Walker S., Morrissy C., Wong F.Y., Meehan B. (2015). Detection of highly pathogenic zoonotic influenza virus H5N6 by reverse-transcriptase quantitative polymerase chain reaction. Virol. J..

[25] Hoffmann E., Stech J., Guan Y., Webster R.G., Perez D.R. (2001). Universal primer set for the full-length amplification of all influenza A viruses. Arch. Virol..

[26] Hebert P.D., Stoeckle M.Y., Zemlak T.S., Francis C.M. (2004). Identification of Birds through DNA Barcodes. PLoS Biol..

[27] Ip H.S., Uhm S., Killian M.L., Torchetti M.K. (2023). An Evaluation of Avian Influenza Virus Whole-Genome Sequencing Approaches Using Nanopore Technology. Microorganisms.

[28] Tamura K., Nei M. (1993). Estimation of the number of nucleotide substitutions in the control region of mitochondrial DNA in humans and chimpanzees. Mol. Biol. Evol..

[29] Kumar S., Stecher G., Tamura K. (2016). MEGA7: Molecular Evolutionary Genetics Analysis Version 7.0 for Bigger Datasets. Mol. Biol. Evol..

[30] Bouckaert R., Vaughan T.G., Barido-Sottani J., Duchêne S., Fourment M., Gavryushkina A., Heled J., Jones G., Kühnert D., De Maio N. (2019). BEAST 2.5: An advanced software platform for Bayesian evolutionary analysis. PLoS Comput. Biol..

[31] Shapiro B., Rambaut A., Drummond A.J. (2006). Choosing Appropriate Substitution Models for the Phylogenetic Analysis of Protein-Coding Sequences. Mol. Biol. Evol..

[32] Drummond A.J., Ho S.Y., Phillips M.J., Rambaut A. (2006). Relaxed Phylogenetics and Dating with Confidence. PLoS Biol..

[33] Rambaut A., Drummond A.J., Xie D., Baele G., Suchard M.A. (2018). Posterior Summarization in Bayesian Phylogenetics Using Tracer 1.7. Syst. Biol..

[34] Suchard M.A., Lemey P., Baele G., Ayres D.L., Drummond A.J., Rambaut A. (2018). Bayesian phylogenetic and phylodynamic data integration using BEAST 1.10. Virus Evol..

[35] Yu G. (2020). Using ggtree to Visualize Data on Tree-Like Structures. Curr. Protoc. Bioinform..

[36] Hew L.Y., Isoda N., Takaya F., Ogasawara K., Kobayashi D., Huynh L.T., Morita T., Harada R., Zinyakov N.G., Andreychuk D.B. (2024). Continuous Introduction of H5 High Pathogenicity Avian Influenza Viruses in Hokkaido, Japan: Characterization of Viruses Isolated in Winter 2022–2023 and Early Winter 2023–2024. Transbound. Emerg. Dis..

[37] Smith D.J., Lapedes A.S., de Jong J.C., Bestebroer T.M., Rimmelzwaan G.F., Osterhaus A.D., Fouchier R.A. (2004). Mapping the antigenic and genetic evolution of influenza virus. Science.

[38] Alkie T.N., Lopes S., Hisanaga T., Xu W., Suderman M., Koziuk J., Fisher M., Redford T., Lung O., Josph T. (2022). A threat from both sides: Multiple introductions of genetically distinct H5 HPAI viruses into Canada via both east Asia-Australasia/Pacific and Atlantic flyways. Virus Evol..

[39] Isoda N., Hiono T., Hew Y.L., Takaya F., Nguyen B.L., Kobayashi D., Fujino K., Sakoda Y. (2025). Dynamics of high pathogenicity avian influenza virus infection with multiple introductions in a crow flock in an urban park in Hokkaido, Japan. Comp. Immunol. Microbiol. Infect. Dis..

[40] Nishiura H., Kumagai A., Maeda M.H., Takadate Y., Sakuma S., Tsunekuni R., Mine J., Uchida Y., Miyazawa K. (2025). Pathogenic and Antigenic Analyses of H5N1 High Pathogenicity Avian Influenza Virus Isolated in the 2022/2023 Season from Poultry Farms in Izumi City, Japan. Transbound. Emerg. Dis..

[41] Kaverin N.V., Rudneva I.A., Govorkova E.A., Timofeeva T.A., Shilov A.A., Kochergin-Nikitsky K.S., Krylov P.S., Webster R.G. (2007). Epitope mapping of the hemagglutinin molecule of a highly pathogenic H5N1 influenza virus by using monoclonal antibodies. J. Virol..

[42] Zhang Y., Cui P., Shi J., Chen Y., Zeng X., Jiang Y., Tian G., Li C., Chen H., Kong H. (2023). Key Amino Acid Residues That Determine the Antigenic Properties of Highly Pathogenic H5 Influenza Viruses Bearing the Clade 2.3.4.4 Hemagglutinin Gene. Viruses.

[43] Isoda N., Onuma M., Hiono T., Sobolev I., Hew Y.L., Nabeshima K., Honjyo H., Yokoyama M., Shestopalov A., Sakoda Y. (2022). Detection of New H5N1 High Pathogenicity Avian Influenza Viruses in Winter 2021–2022 in the Far East, Which Are Genetically Close to Those in Europe. Viruses.

