# Supplementary material for: Genetic Diversity of Highly Pathogenic Avian Influenza Viruses Isolated in Hokkaido, Japan, During Winter 2024–2025
Source: Pathogens. 2025 Sep 21;14(9):951. doi: 10.3390/pathogens14090951 (PMC12472925; doi:10.3390/pathogens14090951)
Supplement: Supplementary file 1 [file pathogens-14-00951-s001.zip › pathogens-3802351-supplementary.pdf]

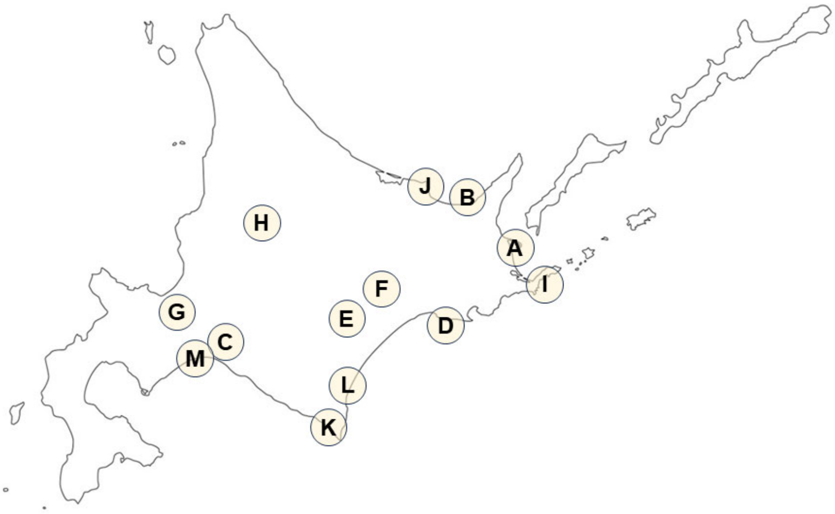

A	Betsukai
B	Shari
C	Atsuma
D	Kushiro
E	Ikeda
F	Honbetsu
G	Sapporo
H	Asahikawa
I	Nemuro
J	Abashiri
K	Erimo
L	Hiroo
M	Tomakomai

Supplemental Figure S1: location of HPAIV positive bird samples found in Hokkaido in winter 2024-2025 season. Dots with alphabet indicate the place and name of city where HPAIV positive bird samples were found.

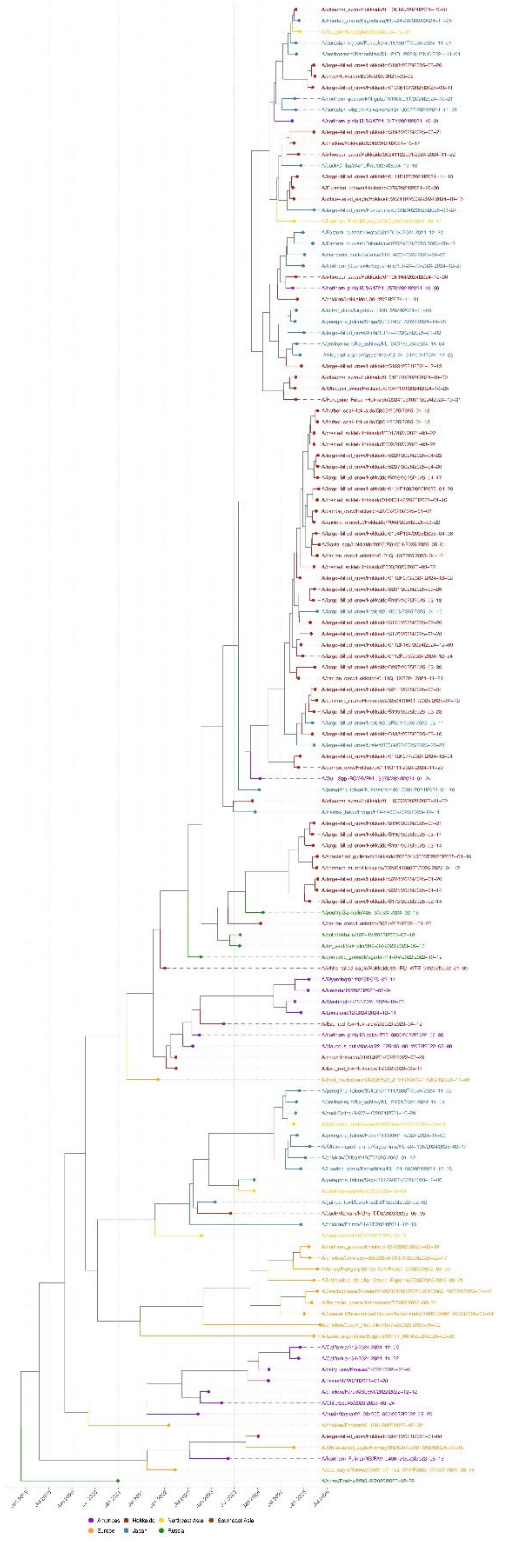

Supplemental Figure S2. Maximum clade credibility (MCC) tree reconstructed from the hemagglutinin gene of H5Nx high pathogenicity avian influenza viruses (clade 2.3.4.4b) collected in Japan (Hokkaido and the main islands) from 2021 to 2025. Same tree with Figure 3 but indicating the name of each strain onto the MCC tree.

Supplemental table S1. Cross-hemagglutination inhibition tests of H5 high pathogenicity avian influenza viruses isolated in Hokkaido in winter 2024-2025

Virus	Clade	Subgroup	Antiserum*													
			Cr/B003/22	Ck/E001/22	Ew/Q71/22	WTE/Hok/22	Md/DRC/17	Ck/Kum/14	Bs/Akita/16	Dk/Vac-1/04	Dk/VTN/23	Dk/Vac-3/07	Pf/HK/09	Md/VTN/11	Ws/Mon/05	Ck/Yama/04
A/large-billed crow/Hokkaido/B003/2022 (H5N2)	2.3.4.4b	G2d	640	640	80	320	1280	640	160	20	640	20	<20	<20	<20	<20
A/chicken/Hokkaido/HU-E001/2022 (H5N1)	2.3.4.4b	G2d	320	640	160	320	1280	640	80	40	640	80	20	20	40	20
A/Eurasian wigeon/Hokkaido/Q71/2022 (H5N1)	2.3.4.4b	G2b	320	640	320	160	1280	320	160	80	640	80	80	40	20	20
A/white-tailed eagle/22-RU-WTE-2/2022 (H5N1)	2.3.4.4b	G2d	640	640	160	320	2560	640	80	40	640	80	20	20	20	20
A/northern pintail/Hokkaido/M13/2020 (H5N8)	2.3.4.4b	G2d	640	640	20	160	2560	640	40	20	640	20	20	<20	<20	<20
A/muscovy duck/DR Congo/KAF1/2017 (H5N8)	2.3.4.4b	-	640	640	80	160	1280	640	80	40	640	20	20	20	20	<20
A/Ezo red fox/Hokkaido/2/2023 (H5N1)	2.3.4.4b	G2d	640	640	160	320	1280	640	80	80	640	40	<20	20	20	20
A/chicken/Hokkaido/HU-B102/2023 (H5N1)	2.3.4.4b	G2c	640	320	80	320	160	160	40	40	1280	80	20	20	40	20
A/large-billed crow/Hokkaido/B067/2023 (H5N1)	2.3.4.4b	G2d	160	160	80	320	320	80	40	40	640	80	<20	20	20	20
A/chicken/Kumamoto/1-7/2014 (H5N8)	2.3.4.4c	G2d	320	640	40	20	640	640	80	20	160	20	<20	<20	<20	<20
A/black swan/Akita/1/2016 (H5N6)	2.3.4.4e	G2d	160	320	20	80	320	160	640	20	160	<20	<20	<20	<20	<20
A/duck/Hokkaido/Vac-1/2004 (H5N1)	Classic	-	<20	20	20	40	40	<20	40	640	20	1280	<20	40	80	40
A/duck/Vietnam/HU16-DD3/2023 (H5N1)	2.3.4.4b	G2c	640	320	80	640	320	320	80	40	640	20	<20	<20	<20	<20
A/duck/Hokkaido/Vac-3/2007 (H5N1)	Classic	-	<20	80	20	160	80	<20	80	1280	20	2560	<20	40	160	160
A/peregrine falcon/Hong Kong/810/2009 (H5N1)	2.3.4	-	20	<20	<20	40	40	<20	40	<20	80	<20	320	<20	<20	<20
A/whooper/swan/Hokkaido/4/2011 (H5N1)	2.3.2.1	-	20	20	<20	<20	80	<20	320	40	80	40	<20	<20	160	<20
A/Muscovy duck/Vietnam/OIE-559/2011 (H5N1)	1	-	40	80	<20	160	80	<20	40	40	40	80	20	640	40	20
A/whooper swan/Mongolia/3/2005 (H5N1)	2.5	-	<20	<20	<20	<20	<20	<20	20	80	20	80	<20	20	160	80
A/chicken/Yamaguchi/7/2004 (H5N1)	2.5	-	20	40	20	80	40	<20	80	320	80	160	<20	40	640	1280
A/large-billed crow/Hokkaido/B073/2024 (H5N5)	2.3.4.4b	G2a	640	160	40	640	640	320	320	20	320	20	<20	20	20	<20
A/Ezo red fox/Hokkaido/1/2022 (H5N1)	2.3.4.4b	G2d	640	320	160	640	640	320	160	40	640	40	<20	20	20	<20
A/peregrine falcon/Saga/4112A002/2023 (H5N6)	2.3.4.4b	G2c	160	160	80	640	160	320	160	<20	160	20	<20	<20	<20	<20
A/large-billed crow/Hokkaido/B147/2024 (H5N1)	2.3.4.4b	G2d	640	320	160	640	640	320	80	80	320	80	640	40	20	320
A/chicken/Hokkaido/J001/2024 (H5N1)	2.3.4.4b	G2d	1280	640	160	1280	1280	640	80	80	640	80	640	40	<20	160
A/large-billed crow/Hokkaido/B169/2024 (H5N1)	2.3.4.4b	G2d	1280	640	160	1280	1280	640	80	80	640	80	1280	40	<20	160
A/Eurasian wigeon/Hokkaido/Q76/2024 (H5N1)	2.3.4.4b	G2d	640	320	160	1280	1280	640	40	80	640	40	640	20	20	160
A/harbor seal/Hokkaido/Q001/2025 (H5N1)	2.3.4.4b	G2d	1280	640	160	1280	1280	640	40	40	1280	80	1280	40	<20	320
A/large-billed crow/Hokkaido/B172/2024 (H5N1)	2.3.4.4b	G2d	1280	640	160	1280	1280	640	40	40	640	40	640	20	<20	160
A/chicken/Hokkaido/E002/2024 (H5N1)	2.3.4.4b	G2d	640	640	160	1280	640	640	40	40	640	40	640	20	<20	80

*: Each of abbreviation name is as followings: Cr/B003/22: A/large-billed crow/Hokkaido/B003/2022 (H5N2), Ck/E001/22: A/chicken/Hokkaido/HU-E001/2022 (H5N1), Ew/Q71/22: A/Eurasian wigeon/Hokkaido/Q71/2022 (H5N1), WTE/Hok/22: A/white-tailed eagle/22-RU-WTE-2/2022 (H5N1), Np/M13/20: A/northern pintail/Hokkaido/M13/2020 (H5N8), Md/DRC/17: A/muscovy duck/DR Congo/KAF1/2017 (H5N8), Ck/Kum/14: A/chicken/Kumamoto/1-7/2014 (H5N8), Bs/Akita/16: A/black swan/Akita/1/2016 (H5N6), Dk/Vac-1/04: A/duck/Hokkaido/Vac-1/2004 (H5N1), Dk/VTN/23: A/duck/Vietnam/HU16-DD3/2023 (H5N1), Dk/Vac-3/07: A/duck/Hokkaido/Vac-3/2007 (H5N1), Pf/HK/09: A/peregrine falcon/Hong Kong/810/2009 (H5N1), Ws/Mon/05: A/whooper swan/Mongolia/3/2005 (H5N1), Ck/Yama/04: A/chicken/Yamaguchi/7/2004 (H5N1)
